# Supplementary material for: Molecular basis of MKLP2-dependent Aurora B transport from chromatin to the anaphase central spindle
Source: J Cell Biol. 2020 May 1;219(7):e201910059. doi: 10.1083/jcb.201910059 (PMC7337490; doi:10.1083/jcb.201910059)
Supplement: Table S1 — lists data collection and refinement statistics for structures. [file JCB_201910059_TableS1.docx]

|  | **CPI^NT^** | **CPI58–H3pT3** | **CPI80–H3pT3** | **MKLP2^596-668^** |
| --- | --- | --- | --- | --- |
| **Data collection** |  |  |  |  |
| PDB accession number | 6YIE | 6YIF | 6YIH | 6YIP |
| Beamline | PETRA III P14 | Diamond I24 | ESRF ID23-1 | Diamond I24 |
| Space group | P *21 21 21* | P *65* | P *65* | P *21 21 21* |
| a, b, c, (Å) | 59.54, 78.82, 125.17 | 98.86, 98.86, 56.07 | 99.37, 99.37, 56.22 | 30.24, 32.93, 159.74 |
| α, β, γ (°) | 90.00, 90.00, 90.00 | 90.00, 90.00, 120.00 | 90.00, 90.00, 120.00 | 90.00, 90.00, 90.00 |
| Wavelength (Å) | 0.97630 | 0.96859 | 0.97625 | 0.96862 |
| Resolution (Å) | 78.82 - 3.49 (3.83 - 3.49) | 49.43 - 1.81 (1.86 – 1.81) | 56.22 - 2.55 (2.66 - 2.55) | 79.868 - 1.43  (1.66 - 1.43) |
| *R*_merge_ | 0.16 (0.44) | 0.05 (0.95) | 0.09 (0.77) | 0.17 (1.63) |
| < I / σI > | 3.5 (1.7) | 13.8 (1.4) | 9.2 (1.8) | 9.2 (1.8) |
| CC (1/2) | 0.992 (0.902) | 0.997 (0.544) | 0.992 (0.706) | 0.997 (0.854) |
| Completeness (%) | 88.5 (90.1) | 99.4 (100) | 96.7 (98.0) | spherical:  36.4 (5.3)  ellipsoidal:  87.9 (66.2) |
| Redundancy | 3.7 (3.7) | 4.4 (4.3) | 4.5 (4.6) | 13.7 (14.2) |
|  |  |  |  |  |
| **Refinement** |  |  |  |  |
| Resolution (Å) | 66.70 - 3.50 | 49.43 - 1.81 | 47.07 - 2.60 | 32.25 - 1.43 |
| No. reflections | 6881 | 28261 | 9833 | 11043 |
| *R*_work_ / *R*_free_ | 0.27 / 0.32 | 0.18 / 0.22 | 0.23 / 0.26 | 0.22 / 0.32 |
| No. atoms |  |  |  |  |
| Protein | 3585 | 1873 | 1886 | 1306 |
| Ligand / ion | Zn: 2 | Zn: 1  Sulphate: 20 | Zn: 1  Sulphate: 10 | 2-propanol: 4 |
| Water |  | 228 | 42 | 152 |
| *B*-factors |  |  |  |  |
| Wilson B | 66.71 | 25.37 | 53.68 | 13.84 |
| Protein | 64.40 | 42.00 | 80.10 | 23.81 |
| Ligand / ion | Zn: 26.75 | Zn: 28.81  Sulphate: 116.27 | Zn: 57.19  Sulphate: 131.49 | 2-propanol: 33.85 |
| Water |  | 46.81 | 59.25 | 29.95 |
| R.m.s. deviations |  |  |  |  |
| Bond length (Å) | 0.004 | 0.011 | 0.004 | 0.013 |
| Angels (°) | 0.81 | 1.12 | 0.650 | 1.40 |
| Ramachandran statistics  (outliers, allowed, favoured) | 0.00, 2.39, 97.61 | 0.00, 1.84, 98.16 | 0.00, 1.31, 98.69 | 0.00, 0.00, 100.00 |
